# Proteomic analysis of post-nuclear supernatant fraction and percoll-purified membranes prepared from brain cortex of rats exposed to increasing doses of morphine

**DOI:** 10.1186/1477-5956-12-11

**Published:** 2014-02-14

**Authors:** Hana Ujcikova, Adam Eckhardt, Dmytro Kagan, Lenka Roubalova, Petr Svoboda

**Affiliations:** 1 Laboratories of Biochemistry of Membrane Receptors, Institute of Physiology, v.v.i., Academy of Sciences of the Czech Republic, Videnska 1083, Prague 4 14220, Czech Republic; 2 Analysis of Biologically Important Compounds, Institute of Physiology, v.v.i., Academy of Sciences of the Czech Republic, Videnska 1083, Prague 4 14220, Czech Republic

**Keywords:** Morphine, Long-term adaptation, Fore brain cortex, Isolated plasma membranes, Post-nuclear supernatant, 2D electrophoresis

## Abstract

**Background:**

Proteomic analysis was performed in post-nuclear supernatant (PNS) and Percoll-purified membranes (PM) prepared from fore brain cortex of rats exposed to increasing doses of morphine (10–50 mg/kg) for 10 days.

**Results:**

In PNS, the 10 up (↑)- or down (↓)-regulated proteins exhibiting the *largest morphine-induced change* were selected, excised manually from the gel and identified by MALDI-TOF MS/MS: **1**-(gi|148747414, Guanine deaminase), ↑2.5×; **2**-(gi|17105370, Vacuolar-type proton ATP subunit B, brain isoform), ↑2.6×; **3**-(gi|1352384, Protein disulfide-isomerase A3), ↑3.4×; **4**-(gi|40254595, Dihydropyrimidinase-related protein 2), ↑3.6×; **5**-(gi|149054470, N-ethylmaleimide sensitive fusion protein, isoform CRAa), ↑2.0×; **6**-(gi|42476181, Malate dehydrogenase, mitochondrial precursor), ↑1.4×; **7**-(gi|62653546, Glyceraldehyde-3-phosphate dehydrogenase), ↑1.6×; **8**-(gi|202837, Aldolase A), ↑1.3×; **9**-(gi|31542401, Creatine kinase B-type), ↓0.86×; **10**-(gi|40538860, Aconitate hydratase, mitochondrial precursor), ↑1.3×. The identified proteins were of cytoplasmic (**1, 4, 5, 7, 9**), cell membrane (**2**), endoplasmic reticulum (**3**) and mitochondrial (**6, 8, 10**) origin and 9 of them were significantly increased, 1.3-3.6×. The 4 out of 9 up-regulated proteins (**4, 6, 7, 10**) were described as functionally related to oxidative stress; the 2 proteins participate in genesis of apoptotic cell death.

In PM, the 18 up (↑)- or down (↓)-regulated proteins were identified by LC-MS/MS and were of *plasma membrane* [Brain acid soluble protein, ↓2.1×; trimeric Gβ subunit, ↓2.0x], *myelin membrane* [MBP, ↓2.5×], *cytoplasmic* [Internexin, ↑5.2×; DPYL2, ↑4.9×; Ubiquitin hydrolase, ↓2.0×; 60S ribosomal protein, ↑2.7×; KCRB, ↓2.6×; Sirtuin-2, ↑2.5×; Peroxiredoxin-2, ↑2.2×; Septin-11, ↑2.2×; TERA, ↑2.1×; SYUA, ↑2.0×; Coronin-1A, ↓5.4×] and *mitochondrial* [Glutamate dehydrogenase 1, ↑2.7×; SCOT1, ↑2.2×; Prohibitin, ↑2.2×; Aspartate aminotransferase**,** ↓2.2×] origin. Surprisingly, the immunoblot analysis of the same PM resolved by 2D-ELFO indicated that the “active”, morphine-induced pool of Gβ subunits represented just a minor fraction of the total signal of Gβ which was decreased 1.2x only. The dominant signal of Gβ was unchanged.

**Conclusion:**

Brain cortex of rats exposed to increasing doses of morphine is far from being adapted. Significant up-regulation of proteins functionally related to oxidative stress and apoptosis suggests a major change of energy metabolism resulting in the state of severe brain cell “discomfort” or even death.

## Background

Morphine is one of the most effective painkillers. Repeated exposure of experimental animals to morphine results in tolerance to this drug, development of physical dependence and a chronic relapsing disorder – drug addiction [1-5]. Physical dependence contributes to a drug seeking behavior and the continuous drug use with the aim to prevent the onset of unpleasant withdrawal symptoms. To name just few, morphine-induced changes of brain function were associated with alternations of synaptic connectivity [6], neurotransmission [7], specific signaling cascades [8], energy metabolism [9] and stability of protein molecules [10].

Hyper-sensitization or super-activation of adenyl cyclase (AC) activity by prolonged exposure of cultured cells or mammalian organism to morphine has been demonstrated in previous studies of mechanism of action of this drug [1-5] and considered as biochemical basis for development of opiate *tolerance* and *dependence*.

Our previous work on isolated Percoll® membranes (PM) prepared from brain cortex of rats exposed to morphine for 10 days (10–50 mg/kg) indicated a desensitization of G-protein response to μ-OR (DAMGO) and δ-OR (DADLE) stimulation [11] and specific increase of ACI (8x) and ACII (2.5×) isoforms [12]. The κ-OR (U-23554)-stimulated [^35^S] GTPγS binding and expression level of ACIII-IX in PM was unchanged. Behavioral tests of morphine-treated animals indicated that these animals were fully drug-dependent (opiate abstinence syndrome) and developed tolerance to subsequent drug addition (analgesic tolerance - hot-plate and hind paw withdrawal tests). The increase of ACI and ACII was interpreted as a specific compensatory response to prolonged stimulation of brain cortex OR by morphine.

Proteomic analysis represents a useful approach for an investigation of the overall changes of protein composition induced by the short-term or prolonged use of drugs. The aim of our present work was to identify proteins which are significantly altered in brain cortex of rats exposed to the increasing, high doses of morphine for prolonged period of time (10 days). For this aim, the post-nuclear supernatant fraction (PNS) was analyzed because it contains proteins of mitochondrial, endoplasmic reticulum, plasma membrane as well as cytoplasmic origin. In the second part of our work, we extended these studies by analysis of protein composition in membrane fraction isolated in Percoll gradient (PM).

## Results

### Two-dimensional electrophoresis and protein identification in post-nuclear supernatant prepared from brain cortex of control and morphine-treated rats; *analysis by MALDI-TOF MS/MS*

Samples of PNS were extracted in ice-cold aceton/TCA/96% ethanol, resolved by 2D-ELFO in linear IPG strips (pH 3–11) and 10% w/v acrylamide/0.26% w/v bis-acrylamide gels as described in methods and stained with silver or colloidal Coomassie blue. The stained 2D gels were scanned with an imaging densitometer and quantified by PDQuest software.

**Figure 1:**
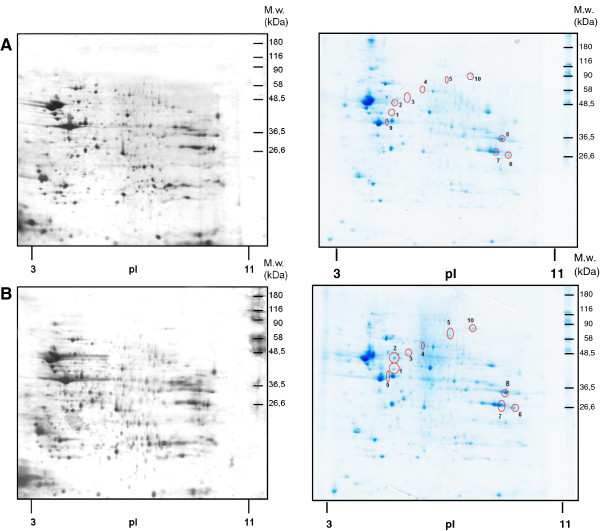
**Two-dimensional gel electrophoresis maps of PNS prepared from control (A) and morphine-treated (B) rats.** Protein samples (600 μg for both Silver and CBB staining) were separated in the first dimension on pH 3–11 IPG strips. For resolution in the second dimension, SDS-PAGE was performed in 10% w/v acrylamide/0.26% w/v bis-acrylamide gel. The stained 2D gels were scanned in an imaging densitometer and quantified by PDQuest software. The process of quantification of the difference between morphine-treated (+M10) and control (−M10) samples included spot detection, gel matching and spot quantification. Master gel was constructed for each group (+M10) or (−M10) as a synthetic image that contains the spot data from all the gels in the MatchSet. At least four replicates were performed for each group/sample. All matched and unmatched spots were then checked in a manual manner. Protein levels altered at least two-fold were taken into consideration. About 200 protein spots totally were recognized by CBB silver staining by PDQuest analysis. Proteins 1–10 with an altered mobility in (+M10) versus (−M10) samples were excised from in CBB-stained gels and identified by MALDI TOF/TOF analyzer as described in methods. *Left panels*, Silver staining; *Right panels*, CBB staining.

About 440 protein spots were recognized by silver staining and PDQuest analysis of gels in both types of PNS (Figure 1, left panels); when stained in colloidal Coomassie blue, about 200 spots were recognized. In CBB-stained gels, proteins 1–10 with different mobility in (+M10) and (−M10) samples were selected for identification by MALDI-TOF MS/MS as described in methods (Figure 1, right panels). The detailed list of the altered and identified proteins is presented in Additional file 1: Table S1 and Table 1. These tables also include description of the subcellular localization and function of these proteins.

**Table 1:** Functional significance of proteins identified in PNS as altered by chronic morphine

**Protein name**	**Change (dependence vs.control)**	**Subcellular localization**	**Functional category**	**Protein characterization - PNS**
Guanine deaminase	Up-regulated	Cytoplasm	Metabolism	Purine metabolism, guanine degradation [13]
V-type proton ATP subunit B, brain isoform	Up-regulated	Cell membrane	Trafficking	ATP hydrolysis coupled proton transport, vacuolar acidification [14]
Protein disulfide-isomerase A3	Up-regulated	Endoplasmatic reticulum lumen	Cellular development and regulation	Up-regulation of this protein causes apoptotic cell death [15], alterations in its level were revealed during neurodegenerative processes [16]
Dihydropyrimidinase-related protein 2	Up-regulated	Cytoplasm	Neuronal development and regulation	Neuronal development and polarity [8], cone collapse and cell migration; one of major determinants in the control of oxidative stress [17]
N-ethylmaleimide sensitive fusion protein, isoform CRA_a	Up-regulated	Cytoplasm	Trafficking	ATP binding, regulating protein membrane trafficking, involved in vesicle priming [18]
Malate dehydrogenase, mitochondrial precursor	Up-regulated	Mitochondrion matrix	Metabolism	L-malate dehydrogenase activity, protein self-association; up-regulation of the mitochondrial malate dehydrogenase is caused by oxidative stress [19]
Glyceraldehyde-3-phosphate dehydrogenase	Up-regulated	Cytoplasm	Metabolism	Glyceraldehyde-3-phosphate dehydrogenase and nitrosylase activities; surprising role in apoptosis [20]; is known as a major target protein in oxidative stress [21]
Aldolase A	Up-regulated	Mitochondrion	Metabolism	Role in glycolysis and gluconeogenesis, scaffolding protein;potential role in regulating the free intracellular concentration of InsP3, and subsequently intracellular calcium dynamics[22,23]; the expression of aldolase A may be regulated by chronic lithium administration [24]
Creatine kinase B-type	Down-regulated	Cytoplasm	Metabolism	Energy-related (skeletal muscle, heart, brain and spermatozoa), brain development [25]
Aconitate hydratase, mitochondrial precursor	Up-regulated	Mitochondrion	Metabolism	Isomerization of citrate to isocitrate via cis-aconitate;an iron-sulfur protein, the particular susceptibility to oxidative damage may be related to the iron-sulfur cluster [4Fe-4S]in its active site [26]

The identified proteins were of cytoplasmic (**1**-Guanine deaminase, ↑2.5×; **4**-Dihydropyrimidinase-related protein 2,↑3.6×; **5**-N-ethylmaleimide sensitive fusion protein, isoform CRAa, ↑2.0×; **7**-Glyceraldehyde-3-phosphate dehydrogenase, ↑1.6×; **9**-Creatine kinase B-type, ↓0.86), cell membrane (**2**-Vacuolar-type proton ATPase, subunit B, brain isoform), ↑2.6x), endoplasmic reticulum (**3**-Protein disulfide-isomerase A3, ↑3.4×) and mitochondrial (**6**-Malate dehydrogenase, mitochondrial precursor, ↑1.4x; **8**-Aldolase A, ↑1.3×; **10**-Aconitate hydratase, mitochondrial precursor, ↑1.3x) origin. The 9 of them were significantly increased, 1.3-3.6x. Correlation with functional properties of these proteins indicated up-regulation of proteins related to guanine degradation (**1**), vacuolar acidification (**2**), apoptotic cell death (**3**), oxidative stress (**4, 6, 7, 10**), membrane traffic (**5**) and glycolysis (**8**). All together, the spectrum of the altered proteins suggests a major alternation of brain cortex tissue when exposed to increasing doses of morphine. *The most significant change from functional point of view was up-regulation of proteins related to oxidative stress (see * discussion *for further details).*

### Two-dimensional electrophoresis and protein identification in Percoll-purified membranes isolated from brain cortex of control and morphine-treated rats; *analysis by LC-MS/MS*

**Figure 2:**
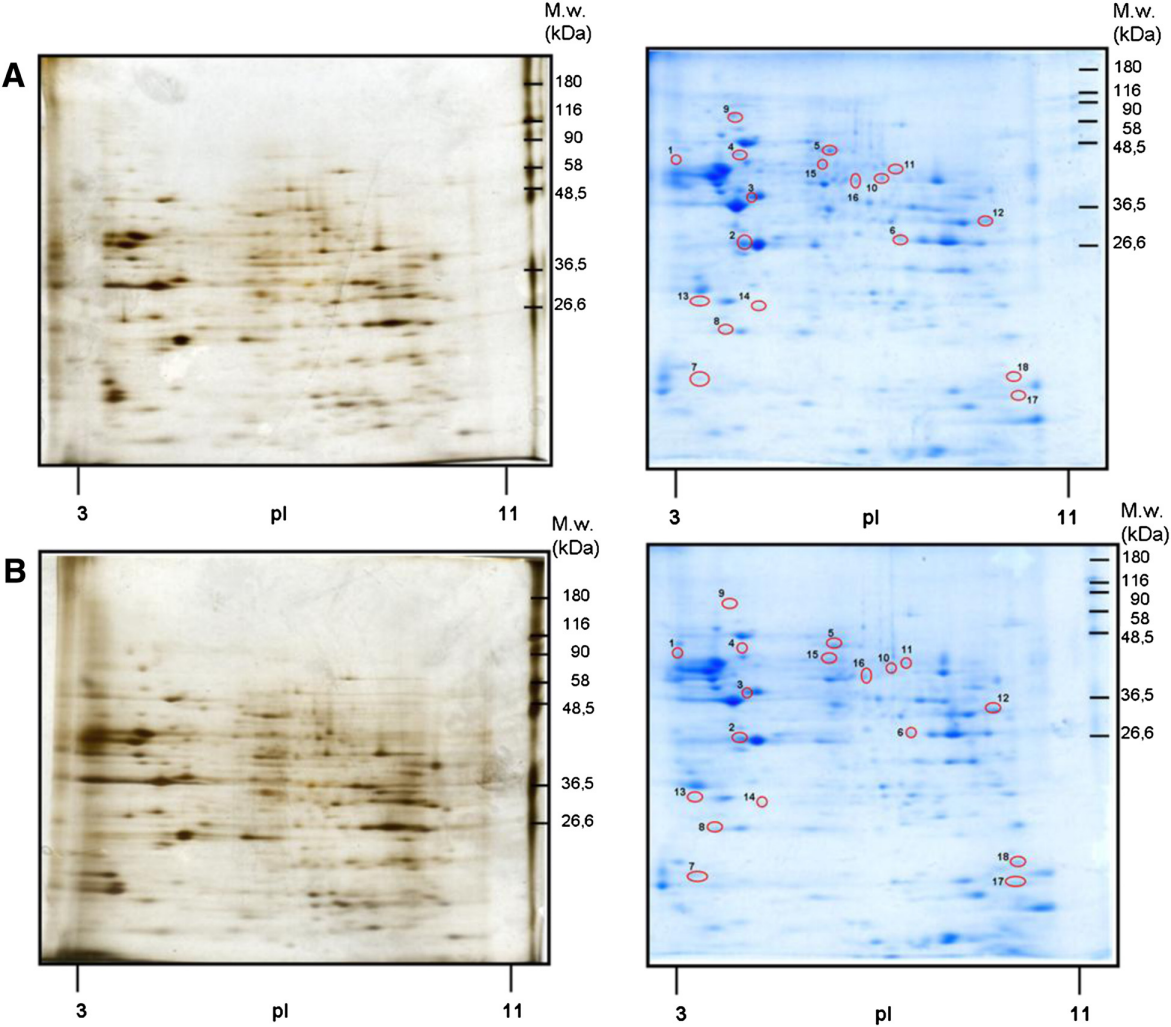
**Two-dimensional gel electrophoresis maps of protein extracts prepared from PM of control (A) and morphine-treated (B) rats.** Protein samples (400 μg for Silver staining; 2 mg for CBB staining) were separated in the first dimension on pH 3–11 IPG strips. For resolution in the second dimension, SDS-PAGE was performed in 10% w/v acrylamide/0.26% w/v bis-acrylamide gel (silver staining) or in 12.5% w/v acrylamide/0.0625% w/v bis-acrylamide gel (CBB staining). The stained 2D gels were scanned in an imaging densitometer and quantified by PDQuest software. The process of quantification of the difference between morphine-treated (+M10) and control (−M10) samples included spot detection, gel matching and spot quantification. Master gel was constructed for each group (+M10) or (−M10) as a synthetic image that contains the spot data from all the gels in the MatchSet. At least four replicates were performed for each group/sample. All matched and unmatched spots were then checked in a manual manner. Protein levels altered at least two-fold were taken into consideration. About 500 protein spots totally were recognized by CBB staining by PDQuest analysis. Proteins 1–18 with an altered mobility in (+M10) versus (−M10) samples were excised from in CBB-stained gels and identified by LC-MS/MS as described in methods. *Left panels*, Silver staining. *Right panels*, Coomassie staining.

PM samples were resolved by 2D-electrophoresis in the same way as described for PNS. The resolution in 10% w/v acrylamide/0.26% w/v bis-acrylamide gels was used in the case of silver staining; 12.0% w/v acrylamide/0.32% w/v bis-acrylamide gels were used for staining in CBB. About 300 protein spots were recognized by silver (Figure 2, left panels); when stained in CBB, the total number of detected protein spots was 490 (Figure 2, right panels). Proteins 1–18 with an altered mobility in (+M10) versus (−M10) samples were excised from in CBB-stained gels and identified by LC-MS/MS. The list of altered and identified proteins is presented in Additional file 2: Table S2 and Table 2. These tables also include a brief description of subcellular localization and function of these proteins as well as quantitative estimate of their relative change induced by morphine-treatment.

**Table 2:** Functional significance of proteins identified in PM fraction as altered by chronic morphine

**Protein name**	**Change (dependence vs.control)**	**Subcellular localization**	**Functional category**	**Protein characterization - PM**
Brain acid soluble protein 1	Down-regulated	Cell membrane; Lipid anchor	Neuronal development and regulation	Associated with the membranes of growth cones that form the tips of elongating axons, DNA-dependent, localizes in the membrane raft domain with a cholesterol-dependent manner; changes in the localization during the development of neuronal polarity [27]
Guanine nucleotide-binding protein subunit beta-1	Down-regulated	Cell membrane	Signaling	Gβ1 is required for neural tube closure, neural progenitor cell proliferation and neonatal development [28]; stimulated ACII, ACIV, ACVII, inhibited ACI, ACV/VI, ACVIII [29,30]
Creatine kinase B-type	Down-regulated	Cytoplasm	Metabolism	Energy-related (skeletal muscle, heart, brain and spermatozoa), brain development [25], aging [31]; one of major determinants in the control of oxidative stress [17]
Alpha-internexin	Up-regulated	Cytoplasm	Neuronal development and regulation	Copurifies with intermediate filaments from rat spinal cord and optic nerve, developmental protein involved in morphogenesis of neurons [32]
Dihydropyrimidinase-related protein 2	Up-regulated	Cytoplasm	Neuronal development and regulation	Neuronal development and polarity [8], cone collapse and cell migration; one of major determinants in the control of oxidative stress [17]
NAD-dependent deacetylase sirtuin-2	Up-regulated	Cytoplasm	Cellular development and regulation	Colocalizes with microtubules; NAD-dependent deacetylase, involved in the control of mitotic exit in the cell cycle; up-regulation may protect the brain against incurred oxidative damage [33]
Alpha-synuclein	Up-regulated	Cytoplasm	Neuronal development and regulation	Specifically expressed in neuronal cell bodies and synapses, negative regulation of neuron apoptosis, aging; role in the pathogenesis of Parkinson’s disease [34]
Peroxiredoxin-2	Up-regulated	Cytoplasm	Neuronal development and regulation	Involved in redox regulation of the cell, negative regulation of neuron apoptosis; the relative abundance appears to protect cellular components by removing the low levels of hydroperoxides and peroxinitrites produced as a result of normal cellular metabolism in the cytosol [35]
Transitional endoplasmic reticulum ATPase	Up-regulated	Cytoplasm Nucleus	Cellular development and regulation	Involved in the formation of the transitional endoplasmatic reticulum, necessary for the fragmentation of Golgi stacks during mitosis and for their reassembly after mitosis [36]; interacts with neurofibromin to control the density of dendritic spines [37]
Glutamate dehydrogenase 1, mitochondrial	Up-regulated	Mitochondrion matrix	Metabolism	Glutamate catabolic process, long-term memory, in rat brain the glutamate dehydrogenase reaction operates in the direction of ammonia production [38]
Succinyl-CoA:3-ketoacid-coenzyme A transferase 1, mitochondrial	Up-regulated	Mitochondrion matrix	Metabolism	A mitochondrial ketone body-activating enzyme [39]; brain development, response to drug
Aspartate aminotransferase, mitochondrial	Down-regulated	Mitochondrion matrix	Metabolism	Amino acid metabolism, metabolite exchange between mitochondria and cytosol, fatty acid transport; its activity is related with the maintenance of amino acid homeostasis and might be an indicator of mitochondrial injury [40]
Ubiquitin carboxyl-terminal hydrolase isozyme L1	Down-regulated	Cytoplasm Endoplasmatic reticulum membrane	Deubiquitination Neuronal development and regulation	Involved both in the processing of ubiquitin precursors and of ubiquitinated proteins; the ubiquitination/proteasome pathway involved in synaptic plasticity [41]
Prohibitin	Up-regulated	Mitochondrion inner membrane	Cellular development and regulation	Antiproliferative activity, role in regulating mitochondrial respiration activity and aging, response to drug [42-44]; down-regulation of prohibitin renders neurons more vulnerable to injury and reactive oxygen species production, whereas up-regulation appears to be neuroprotective [45]
Coronin-1A	Down-regulated	Cytoplasm	Cellular development and regulation	Invagination of plasma membrane, forming protrusions of plasma membrane involved in cell locomotion; coronin-1A activity is spatially and temporally regulated by phosphoinositides [46]
Septin-11	Up-regulated	Cytoplasm	Cellular development and regulation	Filament-forming cytoskeletal GTPase, cell division; it is involved in dendritic maturation [47]
Myelin basic protein S	Down-regulated	Myelin membrane	Neuronal development and regulation	Myelination, negative regulation of axonogenesis; morphine exposure colud result in a decreased number of myelinated axons [48]
60S ribosomal protein L12	Down-regulated	Cytoplasm	Regulatory	Binds directly to 26S ribosomal RNA; it accesses the importin 11 pathway as a major route into the nucleus [49]

The identified up (↑)- or down (↓)-regulated proteins were of *plasma membrane* [**1**-BASP1, Brain acid soluble protein 1, ↓2.1×; **2**-GBB1, Guanine nucleotide-binding protein subunit beta-1, ↓2.0×], *myelin membrane* [**17**-MBP, Myelin basic protein S, ↓2.5×], *cytoplasmic* [**3**-KCRB, Creatine kinase B-type (EC 2.7.3.2), ↓2.6x; **4**-AINX, Alpha-internexin, ↑5.2×; **5**-DPYL2, Dihydropyrimidinase-related protein 2, ↑4.9×; **6**-SIRT2, NAD-dependent deacetylase sirtuin-2, ↑2.5×; **7**-SYUA, Alpha-synuclein, ↑2.0×; **8**-PRDX2, Peroxiredoxin-2, ↑2.2×; **9**-TERA, Transitional endoplasmic reticulum ATPase, ↑2.1×; **13**-UCHL1, Ubiquitin carboxyl-terminal hydrolase L1, ↓2.0×; **15**-COR1A, Coronin-1A, ↓5.4×, **16**-SEP11, Septin-11, ↑2.2×; **18**-RL12, 60S ribosomal protein L12, ↑2.7×] and *mitochondrial* [**10**-DHE3, Glutamate dehydrogenase 1, ↑2.7×; **11**-SCOT1, Succinyl-CoA:3-ketoacid-coenzyme A transferase 1, ↑2.2×; **12**-AATM, Aspartate aminotransferase**,** ↓2.2×; **14**-PHB, Prohibitin, ↑2.2×] origin.

**Figure 3:**
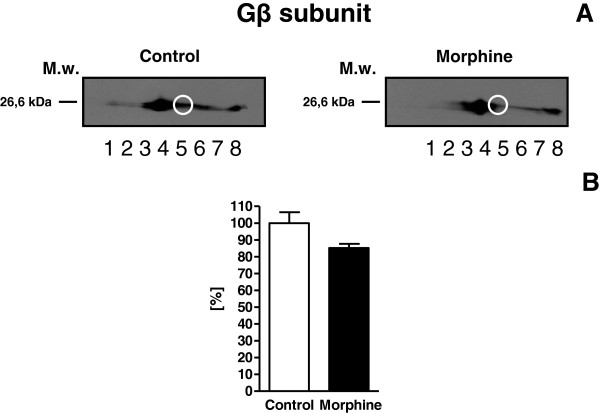
**Gβ subunit protein; *immunoblot analysis of 2D-gels. *A** Two-dimensional resolution of Gβ protein content in PM isolated from control and morphine-adapted rats. PM protein (400 μg) was resolved by 2D electrophoresis using the pI range 3–11 for isoelectric focusing in the first dimension. The white small circle shows the small fraction of the the total signal of Gβ which was taken into consideration when analyzed by LC-MS/MS. The second dimension was performed by SDS-PAGE in 10% w/v acrylamide/0.26% bis-acrylamide gels (Hoefer SE 600). Gβ was identified by immunoblotting with specific antibody as described in Material and methods. The numbers 1–8 represent spots of Gβ subunits which were subsequently analyzed by LC-MS/MS. **B** The average of 3 immunoblots ± SEM. Difference between (−M10) and (+M10) was analyzed by Student’s *t*-test using GraphPad*Prizm4* and found not significant, NS (p > 0.05).

Thus, the only member of GPCR-initiated signaling cascades identified by LC-MS/MS was trimeric Gβ subunit, which was decreased 2× in PM samples prepared from morphine-adapted rats. The morphine-induced decrease of Gβ subunit in PM was subsequently verified by immunoblot analysis of the same 2D-gels as those used for preparation of samples for LC-MS/MS **(**Figure 3). The spot 2 (compare with Figure 2) represented just a small fraction of the total signal of Gβ subunits which was distributed over wider range of pI. The total signal of Gβ was decreased 1.2x only. We have divided the signal of Gβ in CBB-stained gels into 8 small spots according to immunoblot signal (Figure 3) in order to verify it. Proteomic analysis was performed by LC-MS/MS and positive signal was detected in spots 3, 4, 5, 7 and 8 (Table 3).

**Table 3:** Proteomic analysis of Gβ subunits isolated from brain cortex of control and morphine-treated rats

**Spot**	**Accession**	**Protein name**	**Mascot**	**Matched**	**Peptides**	**SC**^**a**^	**MW**^**b**^	**pI**^**c**^
	**number**		**score**	**peptides**		**[%]**	**(kDa)**	
3	GBB1_RAT	**Guanine nucleotide-binding protein**	184.3	6	R.LFDLR.A	16.8	37.4	5.6
		**subunit beta-1**			R.LLVSASQDGK.L			
					K.LWDVR.E			
					K.IYAMHWGTDSR.L + Oxidation (M)			
					K.ACADATLSQITNNIDPVGR.I			
					K.VHAIPLR.S			
4	GBB1_RAT	**Guanine nucleotide-binding protein**	471	12	K.ACADATLSQITNNIDPVGR.I	32.9	37.4	5.6
		**subunit beta-1**			R.LFVSGACDASAK.L			
					K.IYAMHWGTDSR.L + Oxidation (M)			
					R.LFDLR.A			
					K.IYAMHWGTDSR.L			
					R.LLVSASQDGK.L			
					K.LWDVR.E			
					R.KACADATLSQITNNIDPVGR.I			
					R.LLLAGYDDFNCNVWDALK.A			
					K.VHAIPLR.S			
					K.LIIWDSYTTNK.V			
					R.ELAGHTGYLSCCR.F			
4	GBB2_RAT	**Guanine nucleotide-binding protein**	413.9	10	R.TFVSGACDASIK.L	14.4	37.3	5.6
		**subunit beta-2**			K.ACGDSTLTQITAGLDPVGR.I			
					K.IYAMHWGTDSR.L + Oxidation (M)			
					R.LFDLR.A			
					K.IYAMHWGTDSR.L			
					R.LLVSASQDGK.L			
					K.LWDVR.D			
					K.VHAIPLR.S			
					K.LIIWDSYTTNK.V			
					R.LLLAGYDDFNCNIWDAMK.G + Oxidation (W)			
5	GBB1_RAT	**Guanine nucleotide-binding protein**	199.1	5	R.AGVLAGHDNR.V	14.1	37.4	5.6
		**subunit beta-1**			R.LFVSGACDASAK.L			
					R.LFDLR.A			
					K.IYAMHWGTDSR.L + Oxidation (M)			
					R.LLVSASQDGK.L			
5	GBB2_RAT	**Guanine nucleotide-binding protein**	188.7	5	R.AGVLAGHDNR.V	3.5	37.3	5.6
		**subunit beta-2**			R.TFVSGACDASIK.L			
					R.LFDLR.A			
					K.IYAMHWGTDSR.L + Oxidation (M)			
					R.LLVSASQDGK.L			
7	GBB (1–4)_RAT	**Guanine nucleotide-binding protein**	22.4	1	K.LLVSASQDGK.L	2.9	37.2	5.4
		**subunit beta-3**						
8	GBB (1–4)_RAT	**Guanine nucleotide-binding protein**	34.7	1	K.LLVSASQDGK.L	2.9	37.2	5.4
		**subunit beta-3**						

^*a*^Sequence coverage.

^*b*^Theoretical molecular weight.

^*c*^Theoretical isoelectric point.

Therefore, the decrease of Gβ determined by proteomic analysis (2×) has to be regarded as an alternation of relatively small fraction of numerous forms of Gβ resolved by 2D-ELFO. Morphine-induced decrease of Gβ is selectively oriented to specific, minority component of this protein; the dominant pool of Gβ subunits is unchanged.

## Discussion

Opium extracts from the plant Papaver somniferum have been used for therapeutic and recreational purposes for thousands of years. Opioid alkaloids and related pharmaceuticals are the most effective analgesics for the treatment of acute and chronic pain. They also represent one of the largest components of the illicit drug market worldwide, generating revenue of approximately $70 billion in 2009, much of which supports crime, wars and terrorism. Intravenous use of opioid drugs is a leading cause of death by overdose in Europe and North America, and a major contributing factor to the worldwide AIDS epidemic [50,51].

Morphine and codeine are the main active opioid alkaloids in opium. In humans, they act on the central nervous system to produce a wide range of effects including analgesia, euphoria, sedation, respiratory depression and cough suppression. Chronic opiate administration results in the development of tolerance and dependence, but the regulation of MOR and DOR function during this process is not clearly understood.

To localize changes of MOR-stimulated G-protein activity in various brain regions after chronic morphine treatment, Sim et al. [52] examined [^35^S]GTPγS binding to brain sections by in vitro autoradiography. Rats were treated for 12 d with increasing doses (10–320 mg . kg^-1^ . d^-1^) of morphine. Control rats were injected with either saline or a single acute injection of morphine (20 mg/kg). [^35^S]GTPγS binding was measured in the presence or absence of MOR-selective agonist DAMGO. In rats injected with a *single acute dose* of morphine, no significant changes were detected in basal or DAMGO-stimulated [^35^S]GTPγS binding in any brain region. In *chronic morphine-treated rats*, however, DAMGO-stimulated [^35^S]GTPγS binding in cerebral cortex was significantly decreased when compared with control rats. Similar data were obtained in analysis of MOR-stimulated [^35^S]GTPγS binding after chronic heroin administration [53,54]. Accordingly, our analysis of PM isolated from cerebral cortex of rats exposed to morphine for 10 days (10–50 mg/kg) indicated significant desensitization of G-protein response to MOR and DOR stimulation [11] and up-regulation of ACI and II [12].

Proteome changes after prolonged morphine exposure have been so far investigated in “frozen tissue powders” of the rat cerebral cortex, hippocampus, striatum [55,56] and nucleus accumbens [7] or in the “whole-cell lysates” of striatal neuronal cell cultures [57]. Therefore, the aim of our work was to perform proteomic analysis in *more defined* preparations: post-nuclear supernatant (PNS) and membranes isolated in Percoll® gradient (PM). The morphine- induced changes in protein composition (proteom) of PNS and PM were determined by 2D-electrophoresis resolution and PDQuest analysis; the altered proteins were identified by MALDI-TOF MS/MS or LC-MS/MS.

Proteomic analysis of PNS indicated a marked increase of proteins of mitochondrial and cytoplasmic origin (Additional file 1: Table S1 and Table 1). The 9 out of 10 proteins exhibiting the largest morphine-induced change in Coomassie stained gels were increased *by* morphine: **1**-Guanine deaminase, ↑2.5×; **2**-Vacuolar-type proton ATP subunit B, brain isoform ↑2.6×; **3**-Protein disulfide-isomerase A3, ↑3.4×; **4**-Dihydropyrimidinase-related protein 2, ↑3.6x; **5**-N-ethylmaleimide sensitive fusion protein, isoform CRAa, ↑2.0×; **6**-Malate dehydrogenase, mitochondrial precursor, ↑1.4×; **7**-Glyceraldehyde-3-phosphate dehydrogenase, ↑1.6×; **8**-Aldolase A, ↑1.3×; **10**-Aconitate hydratase, mitochondrial precursor, ↑1.3×**.** The 4 out of 9 up-regulated proteins (**4**, **6**, **7**, **10**) were described as functionally related to manifestation of oxidative stress conditions [17,19,21,26]. Marked increase of Protein disulfide-isomerase A3 (**3**) causing apoptotic cell death [15] should be also noticed. The role in apoptosis has been also described for Glyceraldehyde-3-phosphate dehydrogenase (**7**), already mentioned as major target protein in oxidative stress [21]. The 4 out of 9 up-regulated proteins (**4**, **6**, **7**, **10**) were thus functionally related to manifestation of the state of oxidative stress/oxidative damage in brain of morphine-exposed rats and 2 proteins were related to apoptotic cell death.

More detailed analysis of Percoll-purified membranes indicated a complex reorganization of PM protein composition. The list of proteins presented in Additional file 2: Table S2 and Table 2 indicates that morphine-induced alternation included increase as well as decrease of wide range of proteins functionally and structurally related to plasma, myelin, endoplasmic reticulum and mitochondrial membranes. Numerous soluble enzymes present in soluble, cytosol fraction or in mitochondrial matrix were also altered by chronic morphine. Surprisingly, with the exception of trimeric Gβ subunit, not just one of these proteins was functionally related to GPCR- or ionic-channel-activated signaling cascades. Similarly, proteomic analysis of protein alternations induced in the long-term TRH-treatment of HEK cells expressing TRH-R and G11α protein indicated the change of 42 proteins, but not even one of these proteins represented plasma membrane protein functionally related to GPCR-initiated signaling cascades [58].

Our results indicate that the energy metabolism of rat brain cortex exposed to increasing doses of morphine (10–50 mg/kg, 10 days) is shifted far from the normal, physiological state. Using other words, brain cortex of rats exposed to morphine according to our protocol is far from being adapted. It may be suggested that the both neuronal and glial cells undergo a drastic reorganization as consequence of cell discomfort and, subsequently, oxidative stress. Simultaneous activation of all types of opioid receptors (μ-, δ- and κ-OR) by high doses of morphine results in high energy demand of neurons [59,60]. Consequently, glycogen in astrocytes as the single largest energy reserve in the brain is mobilized with the aim to match these increased energy requirements [61]. After depletion of glycogen in astrocytes, the state of oxidative stress appears [62] as the full supply of oxygen to brain mitochondria is not accompanied by transfer of the sufficient number of “reducing equivalents” into the mitochondrial matrix.

## Conclusions

Proteomic analysis of rat brain cortex of rats exposed to morphine for 10 days (10–50 mg/kg) indicated a significant morphine-induced change of membrane protein composition. Changes in *post-nuclear supernatant* were exclusively based on increase (1.3-3.6×) of proteins of mitochondrial and cytoplasmic origin. In isolated *plasma membranes* (PM), morphine-induced alternation included increase as well as decrease of wide range of proteins functionally and structurally related to plasma, myelin, endoplasmic reticulum and mitochondrial membranes. Numerous soluble enzymes present in soluble, cytosol fraction or in mitochondrial matrix were also altered by chronic morphine. The only member of GPCR-initiated signaling cascades identified by LC-MS/MS in Percoll-purified membranes was trimeric Gβ subunit (**2**-GBB) which was decreased 2x in samples prepared from morphine-adapted rats. This “active” component of Gβ subunits, however, represented a minor pool of total complement of Gβ molecules in PM, which was unchanged.

## Material and methods

### Chemicals

Acrylamide, bis-acrylamide and Coomassie Blue G-250 were from SERVA (Heidelberg, Germany), nitrocellulose membrane was from Whatman (Germany). Immobiline Dry-Strips, Pharmalyte buffer, and secondary anti-rabbit antibody labeled with horseradish peroxidase were purchased from GE Healthcare (Piscataway, NJ). Complete protease inhibitor cocktail was from Roche Diagnostic, Mannheim, Germany (cat. no. 1697498). All others chemicals were from Sigma-Aldrich and were of highest purity available. Primary antibody oriented against trimeric Gβ subunit protein (T-20, sc-378) was from Santa Cruz.

### Animals

Male Wistar rats (220-250 g) were killed by decapitation under ether narcosis, the frontal brain was rapidly removed, washed intensively from the remaining blood and cooled to 0ºC. The cerebral cortex was separated on the pre-cooled plate, snap frozen in liquid nitrogen and stored at −70°C until use. The experiments were approved by Animal Care and Use Committee of the Institute of Physiology, Academy of Sciences of the Czech Republic to be in agreement with Animal Protection Law of the Czech Republic as well as European Comunity Council directives 86/609/EEC.

### Morphine treatment of experimental animals

The animals were exposed to morphine by intra-muscular application according to the following protocol: 10 mg/kg (day 1 and 2), 15 mg/kg (day 3 and 4), 20 mg/kg (day 4 and 5), 30 mg/kg (day 6 and 7), 40 mg/kg (day 9) and, finally 50 mg/kg (day 10). The *morphine-adapted* rats were sacrificed 24 hours after the last dose of the drug (group + M10). Control animals were injected with sterile PBS and sacrificed in parallel with morphine-treated rats, i.e. 24 hours (group − M10) after the last dose [12].

### Subcellular fractionation of rat brain cerebral cortex; preparation of post-nuclear supernatant (PNS) and percoll-purified membranes (PM)

Rat brain cortex was minced with razor blade on pre-cooled plate and diluted in STEM medium containing 250 mM sucrose, 20 mM Tris–HCl, 3 mM MgCl_2_, 1 mM EDTA, pH 7.6, fresh 1 mM PMSF plus protease inhibitor cocktail. It was then homogenized mildly in loosely-fitting Teflon-glass homogenizer for 5 min (2 g w. w. per 10 ml) and centrifuged for 5 min at 3500 rpm (1200 × *g*). Resulting post-nuclear supernatant (PNS) was filtered through Nylon nets of decreasing size (330, 110 and 75 mesh, Nitex) and applied on top of Percoll in Beckman Ti70 tubes (30 ml of 27.4% Percoll in STE medium). Centrifugation for 60 min at 30000 rpm (65000 × *g*) resulted in the separation of two clearly visible layers (Bourova et al., 2009). The upper layer represented plasma membrane fraction (PM); the lower layer contained mitochondria (MITO). The upper layer was removed, diluted 1:3 in STEM medium and centrifuged in Beckman Ti70 rotor for 90 min at 50000 rpm (175000 × *g*). Membrane sediment was removed from the compact, gel-like sediment of Percoll and re-homogenized by hand in a small volume of 50 mM Tris–HCl, 3 mM MgCl_2_, 1 mM EDTA, pH 7.4 (TME medium).

### SDS-PAGE and immunoblotting

The aliquots of membrane fractions were mixed 1:1 with 2x concentrated Laemmli buffer (SLB) and heated for 3 min at 95 °C. Standard (10% w/v acrylamide/0.26% w/v bis-acrylamide) SDS electrophoresis was carried out as described before [63-65]. Molecular mass determinations were based on pre-stained molecular mass markers (Sigma, SDS 7B). After SDS-PAGE, proteins were transferred to nitrocellulose and blocked for 1 h at room temperature in 5% (w/v) low-fat milk in TBS-Tween buffer [10 mM Tris–HCl, pH 8.0, 150 mM NaCl, 0.1% (v/v) Tween 20]. Antibodies were added in TBS-Tween containing 1% (w/v) low-fat milk and incubated for at least 2 h. The primary antibody was then removed and the blot washed extensively (3x10 min) in TBS-Tween. Secondary antibodies (donkey anti-rabbit IgG conjugated with horse-radish peroxidase) were diluted in TBS-Tween containing 1% (w/v) low-fat milk, applied for 1 h and after three 10 min washes, the blots were developed by ECL technique using Super Signal West Dura (Pierce) as substrate. The developed blots were scanned with an imaging densitometer ScanJett 5370C (HP) and quantified by Aida Image Analyzer v. 3.28 (Ray test).

### Sample preparation for isoelectric focusing

Samples of PNS or PM containing 400–600 μg protein or 2 mg protein, respectively, were precipitated with ice cold aceton overnight at – 20°C. After centrifugation at 16 000 × *g* for 20 min at 4˚C, the supernatant was removed and the pellet was precipitated with ice-cold 6% TCA for 1.5 h on ice. After centrifugation at 16 000 × *g* for 10 min at 4˚C, the supernatant was discarded and the pellet washed with 400 μl of ice-cold 96% ethanol for 1 h at room temperature. The mixture was centrifuged at 16 000 × *g* for 10 min at 4˚C and the remaining pellet was solubilizated with 250 μl IEF sample buffer containing 7 M urea, 2 M thiourea, 4% CHAPS, 1% DTT, 1% ampholines pH 3–10 and 0.01% bromphenol blue for 3 h at room temperature. After a brief centrifugation (16 000 × *g*, 1 min), the sample was transferred into a groove of the Immobiline DryStrip Reswelling Tray (GE Healthcare).

### Two-dimensional electrophoresis (2D-ELFO)

Immobiline DryStrips (linear pH gradient 3–11 NL, 13 cm) were placed into the Immobiline DryStrip Reswelling Tray containing protein samples and rehydrated overnight.

Isoelectric focusing was performed using the Multiphor II system (GE Healthcare) at 15˚C in the following manner: 150 V for 5 h, 500 V for 1 h, 3500 V for 12 h and 500 V for 3 h. The focused strips were stored at – 20˚C or immediately used.

Strips were rinsed thoroughly with ultrapure water, dried quickly on filter paper and equilibrated in 4 ml of equilibration buffer (50 mM Tris–HCl pH 6.8, 6 M urea, 0.1 mM EDTA, 2% SDS, 30% glycerol and 0.01% bromophenol blue) containing 1% DTT for 15 min in order to reduce disulphide bridges and other oxidized groups. Subsequently, the strips were alkylated in equilibration buffer containing 2.5% iodoacetamide for 15 min. Molecular weight markers were loaded onto a piece of filter paper and placed close to the alkaline side of the strip. The strip and molecular marker were covered with 0.5% agarose. Gels were run vertically at a constant current of 10 mA for 20 min and then at 80 mA for 2 h till the bromophenol blue dye reached the end of the gel. The apparatus was cooled to 15˚C using the Hoefer SE 600 unit (GE Healthcare).

### Silver staining

Silver staining was performed by ProteoSilver™ Plus Silver Stain Kit (Sigma-Aldrich) according to the manufacturer’s instructions [66-68]. Briefly, the gel was fixed in 40% ethanol/10% acidic acid overnight and then washed by 30% ethanol for 10 min and once by ultrapure water for 10 min. The gels were incubated for 10 min with 1% Sensitizer solution and washed twice with 200 ml of ultrapure water for 10 min. The gels were submerged in 1% Silver solution for 10 min, washed with 200 ml of ultrapure water for 1 min and developed with 100 ml of the Developer solution until the desired intensity of spots was attained. The ProteoSilver Stop solution was added to the Developer solution and gels were incubated for 5 min. All steps were carried out at room temperature on an orbital shaker at 60 to 70 rpm. The gels were stored in fresh, ultrapure water or dried in 3% glycerol/25% methanol.

### Colloidal coomassie staining

For MS analysis, the gels were stained by colloidal Coomassie Blue G-250 [69]. The gel was fixed in 40% methanol/5% orthophosphoric acid for 12 h and incubated with colloidal Coomassie Blue (17% ammonium sulphate, 34% methanol, 3% orthophosphoric acid and 0.1% Coomassie G-250) for 48 h. After staining, the gels were kept in 1% acetic acid at 4˚C.

### Image analysis

The stained 2D gels were scanned with an imaging densitometer ScanJett 5370C (HP) and quantified by PDQuest software (Bio-Rad, version 7.3.1). The process included spot detection, gel matching and spot quantification. Master gel was constructed as a synthetic image that contains the spot data from all the gels in the MatchSet. At least four replicates were performed for each sample. All matched and unmatched spots were then checked in a manual manner. Protein levels altered at least two-fold were taken into consideration.

### Preparation of samples for MALDI-TOF MS/MS; *analysis of post-nuclear fraction*

Mass spectrometric analysis MALDI-TOF was performed as described before [58]. The peak lists from the MS spectra were generated by 4000 Series Explorer V 3.5.3 (Applied Biosystems/MDS Sciex) without smoothing, peaks with local signal to noise ratio greater than 5 were picked and searched by local Mascot v. 2.1 (Matrix Science) against nonredundant NCBI database of protein sequences (11186807 sequences; 3815639892 residues). Database search criteria were as follows-enzyme: trypsin, taxonomy: *Rattus norvegicus* (66703 sequences), fixed modification: carbamidomethylation, variable modification: methionine oxidation, peptide mass tolerance: 120 ppm, one missed cleavage allowed. Only hits that were scored as significant (P < 0.001) were included.

### In-gel digestion and preparation of samples for LC-MS/MS; *analysis of percoll-purified membranes (PM)*

Protein spots (from 2-DE: ca 1–2 mm in diameter) were excised from the Coomassie-stained gels, and then processed as described by Shevchenko et al. [70]. Briefly, the spots were first destained by incubation in 100 μl of 100 mM ammonium bicarbonate/acetonitrile (1:1, v/v) with occasional shaking for 1 hour. After destaining, the gel pieces were shrunk by dehydration in 500 μl of acetonitrile, which was then removed and the gel pieces were dried in a vacuum centrifuge. In further step, 100 μl of 10 mM DTT in 100 mM ammonium bicarbonate was added, and the proteins were reduced for 1 hour at 56°C. After cooling to room temperature, the DTT solution was replaced by roughly the same volume of 55 mM iodoacetamide in 100 mM ammonium bicarbonate, and the gels were incubated at ambient temperature for 45 min in the dark. Then the gel pieces were washed with 100 μl of 100 mM ammonium bicarbonate, and dehydrated by addition 500 μl of acetonitrile. Subsequently, the liquid phase was removed and the gel pieces were dried in a vacuum centrifuge.

Before the in-gel digestion, the gel pieces were cooled in an ice-cold bath and swollen in a 100 μl of digestion buffer containing trypsin (20 μg/ml) in 50 mM ammonium bicarbonate, and the gel pieces were sonicated (5 min), placed to air circulation thermostat, and incubated overnight at 37°C. The volumes of solutions needed for processing of the protein bands were four-fold larger than the volumes for processing of the spots. The supernatant of each spot was then transferred to a new vial. The in-gel digestion was performed once more the same way. The resulting tryptic peptides were extracted with sonication (15 min) by 150 μl of extraction buffer (5% formic acid/acetonitrile, 1:2, v/v). Then the solution was spun, the supernatants were transferred, pooled and concentrated to dryness by lyophilization. Dried extracts were stored at −80°C before analysis.

### Analysis of tryptic digests with LC-MS/MS

Dried protein digests were dissolved in 20 μl of 1% formic acid, centrifuged (10 000 × *g*, 5 min, 4°C) and the supernatant transferred to inserts in vials. The nano-HPLC apparatus used for protein digests analysis was a Proxeon Easy-nLC (Proxeon, Odense, Denmark) coupled to a maXis Q-TOF (quadrupole – time of flight) mass spectrometer with ultrahigh resolution (Bruker Daltonics, Bremen, Germany) by nanoelectrosprayer. The nLC-MS/MS instruments were controlled with the software packages HyStar 3.2 and micrOTOF-control 3.0. The data were collected and manipulated with the software packages ProteinScape 2.0 and DataAnalysis 4.0 (Bruker Daltonics).

The 3 μl of the peptide mixture were injected into a NS-AC-11-C18 Biosphere C18 column (particle size: 5 *μ*m, pore size: 12 nm, length: 150 mm, inner diameter: 75 *μ*m), with a NS-MP-10 Biosphere C18 pre-column (particle size: 5 *μ*m, pore size: 12 nm, length: 20 mm, inner diameter: 100 *μ*m), both manufactured by NanoSeparations (Nieuwkoop, Netherlands).

The separation of peptides was achieved via a linear gradient between mobile phase A (water) and B (acetonitrile), both containing 0.1% (v/v) formic acid. Separation was started by running the system with 5% mobile phase B, followed by gradient elution to 30% B at 70 min. The next step was gradient elution to 50% B in 10 min, and then a gradient to 100% B in 8 min was used. Finally, the column was eluted with 100% B for 2 min. Equlibration before the next run was achieved by washing the column with 5% mobile phase B for 10 min. The flow rate was 0.25 *μ*l min^-1^, and the column was held at ambient temperature (25°C).

On-line nano-electrospray ionization (easy nano-ESI) in positive mode was used. The ESI voltage was set at +4.5 kV, scan time 1.3 Hz. Operating conditions: drying gas (N_2_), 1 l min^-1^; drying gas temperature, 160°C; nebulizer pressure, 0.4 bar. Experiments were performed by scanning from 100 to 2200 *m*/*z*. The reference ion used (internal mass lock) was a monocharged ion of C_24_H_19_F_36_N_3_O_6_P_3_ (*m*/*z* 1221.9906). Mass spectra corresponding to each signal from the total ion current chromatogram were averaged, enabling an accurate molecular mass determination. All LC-MS/MS analyses were done in duplicates.

### Database searching

Data were processed using ProteinScape software. Proteins were identified by correlating tandem mass spectra to SwissProt databases, using the MASCOT searching engine (http://www.matrixscience.com); *Rattus norvegicus* as species. Trypsin was chosen as the enzyme parameter. One missed cleavage was allowed, and an initial peptide mass tolerance of ±10.0 ppm was used for MS and ±0.05 Da for MS/MS analysis. Cysteines were assumed to be carbamidomethylated, proline and lysine to be hydroxylated, serine, threonine and tyrosine to be phosphorylated, and methionine was allowed to be oxidated. All these possible modifications were set to be variable. Monoisotopic peptide charge was set to 1+, 2+ and 3+. The Peptide Decoy option was selected during the data search process to remove false-positive results. Only significant hits (MASCOT score ≥60, http://www.matrixscience.com) were accepted.

### Statistical analysis

In immunoblot assays, the significance of difference between data collected in control and morphine-treated samples was analyzed by Student’s *t*-test by GraphPad*Prizm4*. Results represent the average ± S.E.M.

### Protein determination

The method of Lowry was used for determination of membrane protein. Bovine serum albumin (Sigma, Fraction V) was used as standard. Data were calculated by fitting the data with calibration curve as quadratic equation.

## Abbreviations

AC: Adenylyl cyclase; CBB: Coomassie brilliant blue; d: Day; DAMGO: [(2-D-alanine2-4-methylphenylalanine-5-glycineol)-enkefalin]; DADLE: [(2-D-alanine-5-D-leucine)-enkefalin]; DOR: δ-opioid receptor; DTT: Dithiothreitol; EDTA: Ethylenediamine-tetraacetic acid; ELFO: Electrophoresis; GPCR: G protein-coupled receptor; G proteins: Heterotrimeric guanine nucleotide-binding regulatory proteins; CHAPS: 3-[(3-cholamidopropyl)dimethylammonio]-1-propanesulfonate; DTT: Dithiothreitol; IEF: Isoelectric focusing; KOR: κ-opioid receptor; LC-MS/MS: Liquid chromatography–mass spectrometry; MALDI-TOF MS/MS: Matrix-assisted laser desorption/ionization time-of-flight mass spectrometry; MOR: μ-opioid receptor; PBS: Phosphate-buffered saline; PM: Percoll®-purified membranes; PMSF: Phenylmethylsulfonyl fluoride; PNS: Post-nuclear supernatant; SLB: Sample loading buffer; TBS: Tris-buffered saline; w.w.: Wet weight; TCA: Trichloroacetic acid; TRH-R: Thyrotropin-releasing hormone receptor.

## Competing interests

The authors declare that they have no competing interests.

## Authors’ contributions

HU performed the experiments, analyzed the data and participated in writing the manuscript. AE performed proteomic analysis of plasma membrane proteins by LC-MS/MS. DK and LB were responsible for application of morphine to rats according to experimental protocol described in Methods and prepared membrane fractions by differential or density gradient centrifugation. PS conceived the study, designed the experiments and wrote the manuscript. All authors have read and approved the final manuscript.

## Supplementary Material

Additional file 1: Table S1 Proteomic analysis of post-nuclear supernatant prepared from brain cortex of control and morphine-treated rats. Click here for file

Additional file 2: Table S2 Proteomic analysis of PM fraction isolated from brain cortex of control and morphine-treated rats. Click here for file
