# Supplementary material for: Proteomic analysis of post-nuclear supernatant fraction and percoll-purified membranes prepared from brain cortex of rats exposed to increasing doses of morphine
Source: Proteome Sci. 2014 Feb 14;12:11. doi: 10.1186/1477-5956-12-11 (PMC3936806; doi:10.1186/1477-5956-12-11)
Supplement: Additional file 1: Table S1 — Proteomic analysis of post-nuclear supernatant prepared from brain cortex of control and morphine-treated rats. [file 1477-5956-12-11-S1.doc]

**Table 1**

*Proteomic analysis of post-nuclear supernatant prepared from brain cortex of control and morphine-treated rats.*

| **Spot** | **Accession** | **Protein name** | **Mascot** | **Matched** | **Peptides** | **SC^a^** | **MW^b^** | **pI^c^** | **Change** |
| --- | --- | --- | --- | --- | --- | --- | --- | --- | --- |
|  | **number** |  | **score** | **peptides** |  | **[%]** | **(kDa)** |  | **(fold)** |
| 1 | gi|148747414 | **Guanine deaminase** | 278 | 27 | K.SLYPGYK.N | 68 | 51.4 | 5.5 | ↑ 2.5 |
|  |  |  |  |  | K.YTFPTEK.R |  |  |  |  |
|  |  |  |  |  | R.VKPIVTPR.F |  |  |  |  |
|  |  |  |  |  | R.FVSEMLQK.N |  |  |  |  |
|  |  |  |  |  | R.FVSEMLQK.N + Oxidation (M) |  |  |  |  |
|  |  |  |  |  | K.FLYLGDDR.N |  |  |  |  |
|  |  |  |  |  | K.YTFPTEKR.F |  |  |  |  |
|  |  |  |  |  | R.TPQLALIFR.G |  |  |  |  |
|  |  |  |  |  | K.DFDALLINPR.A |  |  |  |  |
|  |  |  |  |  | R.LATLGGSQALGLDR.E |  |  |  |  |
|  |  |  |  |  | K.EWCFKPCEIR.E |  |  |  |  |
|  |  |  |  |  | K.IVFLEESSQQEK.L |  |  |  |  |
|  |  |  |  |  | R.FQSTDVAEEVYTR.V |  |  |  |  |
|  |  |  |  |  | K.RFQSTDVAEEVYTR.V |  |  |  |  |
|  |  |  |  |  | K.NYTDVYDKNNLLTNK.T |  |  |  |  |
|  |  |  |  |  | R.GTFVHSTWTCPMEVLR.D |  |  |  |  |
|  |  |  |  |  | R.GTFVHSTWTCPMEVLR.D  + Oxidation (M) |  |  |  |  |
|  |  |  |  |  | R.FSLSCTETLMSELGNIAK.T |  |  |  |  |
|  |  |  |  |  | K.IGLGTDVAGGYSYSMLDAIR.R |  |  |  |  |
|  |  |  |  |  | K.IGLGTDVAGGYSYSMLDAIR.R  + Oxidation (M) |  |  |  |  |
|  |  |  |  |  | K.FLYLGDDRNIEEVYVGGK.Q |  |  |  |  |
|  |  |  |  |  | R.EIGNFEVGKDFDALLINPR.A |  |  |  |  |
|  |  |  |  |  | K.TVMAHGCYLSEEELNVFSER.G |  |  |  |  |
|  |  |  |  |  | K.THDLYIQSHISENREEIEAVK.S |  |  |  |  |
|  |  |  |  |  | R.ASDSPIDLFCGDFVGDISEAVIQK.F |  |  |  |  |
|  |  |  |  |  | R.GASIAHCPNSNLSLSSGLLNVLDVLK.H |  |  |  |  |
|  |  |  |  |  | K.NGTTTACYFGTIHTDSSLILAEITDK.F |  |  |  |  |
|  |  |  |  |  |  |  |  |  |  |
| 2 | gi|17105370 | **V-type proton ATP subunit B,** | 601 | 33 | R.EEVPGRR.G | 72 | 56.9 | 5.6 | ↑ 2.6 |
|  |  | **brain isoform** |  |  | R.NYLSQPR.L |  |  |  |  |
|  |  |  |  |  | K.SAIGEGMTR.K |  |  |  |  |
|  |  |  |  |  | R.TPVSEDMLGR.V |  |  |  |  |
|  |  |  |  |  | K.TSCEFTGDILR.T |  |  |  |  |
|  |  |  |  |  | K.NFITQGPYENR.T |  |  |  |  |
|  |  |  |  |  | K.KTSCEFTGDILR.T |  |  |  |  |
|  |  |  |  |  | R.IPQSTLSEFYPR.D |  |  |  |  |
|  |  |  |  |  | K.AVVQVFEGTSGIDAK.K |  |  |  |  |
|  |  |  |  |  | R.YAEIVHLTLPDGTK.R |  |  |  |  |
|  |  |  |  |  | K.RIPQSTLSEFYPR.D |  |  |  |  |
|  |  |  |  |  | R.QIYPPINVLPSLSR.L |  |  |  |  |
|  |  |  |  |  | K.TVSGVNGPLVILDHVK.F |  |  |  |  |
|  |  |  |  |  | K.AVVQVFEGTSGIDAKK.T |  |  |  |  |
|  |  |  |  |  | R.TVYETLDIGWQLLR.I |  |  |  |  |
|  |  |  |  |  | R.YAEIVHLTLPDGTKR.S |  |  |  |  |
|  |  |  |  |  | R.LALTTAEFLAYQCEK.H |  |  |  |  |
|  |  |  |  |  | R.GFPGYMYTDLATIYER.A |  |  |  |  |
|  |  |  |  |  | R.GFPGYMYTDLATIYER.A + Oxidation (M) |  |  |  |  |
|  |  |  |  |  | K.HVLVILTDMSSYAEALR.E |  |  |  |  |
|  |  |  |  |  | K.DHADVSNQLYACYAIGK.D |  |  |  |  |
|  |  |  |  |  | K.HVLVILTDMSSYAEALR.E + Oxidation (M) |  |  |  |  |
|  |  |  |  |  | R.GIVNGAAPELPVPTGGPMAGAR.E  + Oxidation (M) |  |  |  |  |
|  |  |  |  |  | R.KDHADVSNQLYACYAIGK.D |  |  |  |  |
|  |  |  |  |  | K.IPIFSAAGLPHNEIAAQICR.Q |  |  |  |  |
|  |  |  |  |  | R.TVYETLDIGWQLLRIFPK.E |  |  |  |  |
|  |  |  |  |  | K.AVVGEEALTSDDLLYLEFLQK.F |  |  |  |  |
|  |  |  |  |  | R.IYPEEMIQTGISAIDGMNSIAR.G |  |  |  |  |
|  |  |  |  |  | R.IYPEEMIQTGISAIDGMNSIAR.G  + Oxidation (M) |  |  |  |  |
|  |  |  |  |  | R.GPVVLAEDFLDIMGQPINPQCR.I |  |  |  |  |
|  |  |  |  |  | K.DVVDYSEENFAIVFAAMGVNMETAR.F |  |  |  |  |
|  |  |  |  |  | K.SKDVVDYSEENFAIVFAAMGVNMETAR.F |  |  |  |  |
|  |  |  |  |  | K.SDFEENGSMDNVCLFLNLANDPTIER.I |  |  |  |  |
|  |  |  |  |  |  |  |  |  |  |
| 3 | gi|1352384 | **Protein disulfide-isomerase A3** | 277 | 18 | K.FVMQEEFSR.D | 36 | 57.0 | 5.9 | ↑ 3.4 |
|  |  |  |  |  | K.FVMQEEFSR.D + Oxidation (M) |  |  |  |  |
|  |  |  |  |  | R.LAPEYEAAATR.L |  |  |  |  |
|  |  |  |  |  | K.GFPTIYFSPANK.K |  |  |  |  |
|  |  |  |  |  | R.FLQEYFDGNLK.R |  |  |  |  |
|  |  |  |  |  | R.DLFSDGHSEFLK.A |  |  |  |  |
|  |  |  |  |  | R.ELNDFISYLQR.E |  |  |  |  |
|  |  |  |  |  | R.LAPEYEAAATRLK.G |  |  |  |  |
|  |  |  |  |  | K.GFPTIYFSPANKK.L |  |  |  |  |
|  |  |  |  |  | R.FLQEYFDGNLKR.Y |  |  |  |  |
|  |  |  |  |  | R.EATNPPIIQEEKPK.K |  |  |  |  |
|  |  |  |  |  | K.DLLTAYYDVDYEK.N |  |  |  |  |
|  |  |  |  |  | K.MDATANDVPSPYEVK.G + Oxidation (M) |  |  |  |  |
|  |  |  |  |  | K.EYDDNGEGITIFRPLHLANK.F |  |  |  |  |
|  |  |  |  |  | K.TFSHELSDFGLESTTGEIPVVAIR.T |  |  |  |  |
|  |  |  |  |  | R.KTFSHELSDFGLESTTGEIPVVAIR.T |  |  |  |  |
|  |  |  |  |  | K.FIQESIFGLCPHMTEDNKDLIQGK.D |  |  |  |  |
|  |  |  |  |  | K.EYDDNGEGITIFRPLHLANKFEDK.I |  |  |  |  |
|  |  |  |  |  |  |  |  |  |  |
| 4 | gi|40254595 | **Dihydropyrimidinase-related**  **protein 2** | 273 | 20 | K.TIEAHSR.M | 40 | 62.7 | 6.0 | ↑ 3.6 |
|  |  |  |  |  | K.VFNLYPR.K |  |  |  |  |
|  |  |  |  |  | K.SAAEVIAQAR.K |  |  |  |  |
|  |  |  |  |  | K.VFNLYPRK.G |  |  |  |  |
|  |  |  |  |  | R.KPFPDFVYK.R |  |  |  |  |
|  |  |  |  |  | R.MVIPGGIDVHTR.F |  |  |  |  |
|  |  |  |  |  | R.KPFPDFVYKR.I |  |  |  |  |
|  |  |  |  |  | R.MVIPGGIDVHTR.F + Oxidation (M) |  |  |  |  |
|  |  |  |  |  | R.GLYDGPVCEVSVTPK.T |  |  |  |  |
|  |  |  |  |  | K.IVLEDGTLHVTEGSGR.Y |  |  |  |  |
|  |  |  |  |  | R.SITIANQTNCPLYVTK.V |  |  |  |  |
|  |  |  |  |  | R.FQLTDSQIYEVLSVIR.D |  |  |  |  |
|  |  |  |  |  | K.SCCDYSLHVDITEWHK.G |  |  |  |  |
|  |  |  |  |  | K.THNSALEYNIFEGMECR.G |  |  |  |  |
|  |  |  |  |  | K.THNSALEYNIFEGMECR.G + Oxidation (M) |  |  |  |  |
|  |  |  |  |  | R.NLHQSGFSLSGAQIDDNIPR.R |  |  |  |  |
|  |  |  |  |  | K.DRFQLTDSQIYEVLSVIR.D |  |  |  |  |
|  |  |  |  |  | K.IVNDDQSFYADIYMEDGLIK.Q |  |  |  |  |
|  |  |  |  |  | R.DIGAIAQVHAENGDIIAEEQQR.I |  |  |  |  |
|  |  |  |  |  | R.ILDLGITGPEGHVLSRPEEVEAEAVNR.S |  |  |  |  |
|  |  |  |  |  |  |  |  |  |  |
| 5 | gi|149054470 | **N-ethylmaleimide sensitive fusion** | 246 | 26 | K.GEPASGKR.Q | 32 | 80.6 | 6.1 | ↑ 2.0 |
|  |  | **protein, isoform CRA_a** |  |  | K.WGDPVTR.V |  |  |  |  |
|  |  |  |  |  | K.IFDDAYK.S |  |  |  |  |
|  |  |  |  |  | R.KFLALMR.E + Oxidation (M) |  |  |  |  |
|  |  |  |  |  | K.AESLQVTR.G |  |  |  |  |
|  |  |  |  |  | R.AAQSTAMNR.H |  |  |  |  |
|  |  |  |  |  | R.AAQSTAMNR.H + Oxidation (M) |  |  |  |  |
|  |  |  |  |  | K.LLIIGTTSR.K |  |  |  |  |
|  |  |  |  |  | K.MIGFSETAK.C + Oxidation (M) |  |  |  |  |
|  |  |  |  |  | K.LLIIGTTSRK.D |  |  |  |  |
|  |  |  |  |  | K.YVGESEANIR.K |  |  |  |  |
|  |  |  |  |  | R.LLDYVPIGPR.F |  |  |  |  |
|  |  |  |  |  | R.LQILHIHTAR.M |  |  |  |  |
|  |  |  |  |  | K.DIEAMDPSILK.G |  |  |  |  |
|  |  |  |  |  | K.MEIGLPDEKGR.L |  |  |  |  |
|  |  |  |  |  | K.YVGESEANIRK.L |  |  |  |  |
|  |  |  |  |  | K.NFSGAELEGLVR.A |  |  |  |  |
|  |  |  |  |  | K.IAEESNFPFIK.I |  |  |  |  |
|  |  |  |  |  | K.DYQSGQHVMVR.T + Oxidation (M) |  |  |  |  |
|  |  |  |  |  | K.LFADAEEEQRR.L |  |  |  |  |
|  |  |  |  |  | R.VLDDGELLVQQTK.N |  |  |  |  |
|  |  |  |  |  | K.SQLSCVVVDDIER.L |  |  |  |  |
|  |  |  |  |  | R.TPLVSVLLEGPPHSGK.T |  |  |  |  |
|  |  |  |  |  | R.THPSVVPGCIAFSLPQR.K |  |  |  |  |
|  |  |  |  |  | R.THPSVVPGCIAFSLPQRK.W |  |  |  |  |
|  |  |  |  |  | K.NSDRTPLVSVLLEGPPHSGK.T |  |  |  |  |
|  |  |  |  |  |  |  |  |  |  |
| 6 | gi|42476181 | **Malate dehydrogenase,** | 604 | 19 | K.KGEDFVK.N | 62 | 36.1 | 8.9 | ↑ 1.4 |
|  |  | **mitochondrial precursor** |  |  | K.ITPFEEK.M |  |  |  |  |
|  |  |  |  |  | K.HGVYNPNK.I |  |  |  |  |
|  |  |  |  |  | R.ANTFVAELK.G |  |  |  |  |
|  |  |  |  |  | K.MIAEAIPELK.A |  |  |  |  |
|  |  |  |  |  | R.VNVPVIGGHAGK.T |  |  |  |  |
|  |  |  |  |  | K.IFGVTTLDIVR.A |  |  |  |  |
|  |  |  |  |  | R.FVFSLVDAMNGK.E |  |  |  |  |
|  |  |  |  |  | K.GCDVVVIPAGVPR.K |  |  |  |  |
|  |  |  |  |  | K.TIIPLISQCTPK.V |  |  |  |  |
|  |  |  |  |  | K.EGVIECSFVQSK.E |  |  |  |  |
|  |  |  |  |  | K.AGAGSATLSMAYAGAR.F |  |  |  |  |
|  |  |  |  |  | K.AGAGSATLSMAYAGAR.F + Oxidation (M) |  |  |  |  |
|  |  |  |  |  | K.GYLGPEQLPDCLK.G |  |  |  |  |
|  |  |  |  |  | K.VDFPQDQLATLTGR.I |  |  |  |  |
|  |  |  |  |  | K.ETECTYFSTPLLLGK.K |  |  |  |  |
|  |  |  |  |  | K.VAVLGASGGIGQPLSLLLK.N |  |  |  |  |
|  |  |  |  |  | R.LTLYDIAHTPGVAADLSHIETR.A |  |  |  |  |
|  |  |  |  |  | K.GYLGPEQLPDCLKGCDVVVIPAGVPR.K |  |  |  |  |
|  |  |  |  |  |  |  |  |  |  |
| 7 | gi|62653546 | **Glyceraldehyde-3-phosphate** | 458 | 15 | K.VGVNGFGR.I | 48 | 36.0 | 8.4 | ↑ 1.6 |
|  |  | **dehydrogenase** |  |  | K.LTGMAFR.V + Oxidation (M) |  |  |  |  |
|  |  |  |  |  | K.VIPELNGK.L |  |  |  |  |
|  |  |  |  |  | R.VVDLMAYMASKE.- |  |  |  |  |
|  |  |  |  |  | R.GAAQNIIPASTGAAK.A |  |  |  |  |
|  |  |  |  |  | R.VPTPNVSVVDLTCR.L |  |  |  |  |
|  |  |  |  |  | K.LVNNGKPITIFQER.D |  |  |  |  |
|  |  |  |  |  | K.LISWYDNEYGYSNR.V |  |  |  |  |
|  |  |  |  |  | K.IVSNASCTTNCLAPLAK.V |  |  |  |  |
|  |  |  |  |  | R.VIISAPSADAPMFVMGVNHEK.Y |  |  |  |  |
|  |  |  |  |  | R.VIISAPSADAPMFVMGVNHEK.Y  + Oxidation (M) |  |  |  |  |
|  |  |  |  |  | K.LVNNGKPITIFQERDPANIK.W |  |  |  |  |
|  |  |  |  |  | K.RVIISAPSADAPMFVMGVNHEK.Y |  |  |  |  |
|  |  |  |  |  | K.VIHDNFGIVEGLMTTVHAITATQK.T |  |  |  |  |
|  |  |  |  |  | K.VIHDNFGIVEGLMTTVHAITATQK.T  + Oxidation (M) |  |  |  |  |
|  |  |  |  |  |  |  |  |  |  |
| 8 | gi|202837 | **Aldolase A** | 599 | 23 | K.ELADIAHR.I | 74 | 39.7 | 8.3 | ↑ 1.3 |
|  |  |  |  |  | K.AAQEEYIKR.A |  |  |  |  |
|  |  |  |  |  | K.ADDGRPFPQVIK.S |  |  |  |  |
|  |  |  |  |  | M.PHPYPALTPEQK.K |  |  |  |  |
|  |  |  |  |  | M.PHPYPALTPEQKK.E |  |  |  |  |
|  |  |  |  |  | K.ADDGRPFPQVIKSK.G |  |  |  |  |
|  |  |  |  |  | R.LQSIGTENTEENRR.F |  |  |  |  |
|  |  |  |  |  | K.FSNEEIAMATVTALR.R |  |  |  |  |
|  |  |  |  |  | K.FSNEEIAMATVTALR.R + Oxidation (M) |  |  |  |  |
|  |  |  |  |  | K.CPLLKPWALTFSYGR.A |  |  |  |  |
|  |  |  |  |  | K.FSNEEIAMATVTALRR.T |  |  |  |  |
|  |  |  |  |  | K.FSNEEIAMATVTALRR.T + Oxidation (M) |  |  |  |  |
|  |  |  |  |  | R.VNPCIGGVILFHETLYQK.A |  |  |  |  |
|  |  |  |  |  | K.IGEHTPSSLAIVENANVLAR.Y |  |  |  |  |
|  |  |  |  |  | K.YTPSGQSGAAASESLFISNHAY.- |  |  |  |  |
|  |  |  |  |  | K.GVVPLAGTNGETTTQGLDGLSER.C |  |  |  |  |
|  |  |  |  |  | K.VDKGVVPLAGTNGETTTQGLDGLSER.C |  |  |  |  |
|  |  |  |  |  | R.TVPPAVPGVTFLSGGQSEEEASINLNAINK.C |  |  |  |  |
|  |  |  |  |  | R.QLLLTADDRVNPCIGGVILFHETLYQK.A |  |  |  |  |
|  |  |  |  |  | K.ALSDHHVYLEGTLLKPNMVTPGHACTQK.F |  |  |  |  |
|  |  |  |  |  | K.ALSDHHVYLEGTLLKPNMVTPGHACTQK.F + Oxidation (M) |  |  |  |  |
|  |  |  |  |  | R.YASICQQNGIVPIVEPEILPDGDHDLKR.C |  |  |  |  |
|  |  |  |  |  | R.RTVPPAVPGVTFLSGGQSEEEASINLNAINK.C |  |  |  |  |
|  |  |  |  |  |  |  |  |  |  |
| 9 | gi|31542401 | **Creatine kinase B-type** | 287 | 15 | K.FSEVLKR.L | 47 | 43.0 | 5.3 | ↓ 0.86 |
|  |  |  |  |  | R.GIWHNDNK.T |  |  |  |  |
|  |  |  |  |  | K.LLIEMEQR.L |  |  |  |  |
|  |  |  |  |  | K.LLIEMEQR.L + Oxidation (M) |  |  |  |  |
|  |  |  |  |  | R.GFCLPPHCSR.G |  |  |  |  |
|  |  |  |  |  | K.DLFDPIIEDR.H |  |  |  |  |
|  |  |  |  |  | K.VLTPELYAELR.A |  |  |  |  |
|  |  |  |  |  | R.FCTGLTQIETLFK.S |  |  |  |  |
|  |  |  |  |  | K.LAVEALSSLDGDLSGR.Y |  |  |  |  |
|  |  |  |  |  | K.TFLVWINEEDHLR.V |  |  |  |  |
|  |  |  |  |  | R.LGFSEVELVQMVVDGVK.L |  |  |  |  |
|  |  |  |  |  | R.GTGGVDTAAVGGVFDVSNADR.L |  |  |  |  |
|  |  |  |  |  | K.RGTGGVDTAAVGGVFDVSNADR.L |  |  |  |  |
|  |  |  |  |  | K.LRFPAEDEFPDLSSHNNHMAK.V |  |  |  |  |
|  |  |  |  |  | K.NYEFMWNPHLGYILTCPSNLGTGLR.A |  |  |  |  |
|  |  |  |  |  |  |  |  |  |  |
| 10 | gi|40538860 | **Aconitate hydratase,** | 348 | 26 | R.EHAALEPR.H | 41 | 86.1 | 7.9 | ↑ 1.26 |
|  |  | **mitochondrial precursor** |  |  | R.DGYAQILR.D |  |  |  |  |
|  |  |  |  |  | K.EGWPLDIR.V |  |  |  |  |
|  |  |  |  |  | R.VDVSPTSQR.L |  |  |  |  |
|  |  |  |  |  | K.NTIVTSYNR.N |  |  |  |  |
|  |  |  |  |  | R.LNRPLTLSEK.I |  |  |  |  |
|  |  |  |  |  | K.LTGTLSGWTSPK.D |  |  |  |  |
|  |  |  |  |  | K.FNPETDFLTGK.D |  |  |  |  |
|  |  |  |  |  | K.SQFTITPGSEQIR.A |  |  |  |  |
|  |  |  |  |  | K.DFAPGKPLNCIIK.H |  |  |  |  |
|  |  |  |  |  | K.FKLEAPDADELPR.S |  |  |  |  |
|  |  |  |  |  | K.VAMSHFEPSEYIR.Y |  |  |  |  |
|  |  |  |  |  | K.VAMSHFEPSEYIR.Y + Oxidation (M) |  |  |  |  |
|  |  |  |  |  | R.NAVTQEFGPVPDTAR.Y |  |  |  |  |
|  |  |  |  |  | R.WVVIGDENYGEGSSR.E |  |  |  |  |
|  |  |  |  |  | K.DINQEVYNFLATAGAK.Y |  |  |  |  |
|  |  |  |  |  | R.VGLIGSCTNSSYEDMGR.S |  |  |  |  |
|  |  |  |  |  | K.IVYGHLDDPANQEIER.G |  |  |  |  |
|  |  |  |  |  | R.AKDINQEVYNFLATAGAK.Y |  |  |  |  |
|  |  |  |  |  | R.DVGGIVLANACGPCIGQWDR.K |  |  |  |  |
|  |  |  |  |  | K.VAVPSTIHCDHLIEAQLGGEK.D |  |  |  |  |
|  |  |  |  |  | R.DVGGIVLANACGPCIGQWDRK.D |  |  |  |  |
|  |  |  |  |  | R.VAMQDATAQMAMLQFISSGLPK.V |  |  |  |  |
|  |  |  |  |  | K.VAVPSTIHCDHLIEAQLGGEKDLR.R |  |  |  |  |
|  |  |  |  |  | R.NDANPETHAFVTSPEIVTALAIAGTLK.F |  |  |  |  |
|  |  |  |  |  | K.HPNGTQETILLNHTFNETQIEWFR.A |  |  |  |  |

*^a^* Sequence coverage.

*^b^* Theoretical molecular weight.

*^c^* Theoretical isoelectric point.
