# Supplementary material for: Proteomic analysis of post-nuclear supernatant fraction and percoll-purified membranes prepared from brain cortex of rats exposed to increasing doses of morphine
Source: Proteome Sci. 2014 Feb 14;12:11. doi: 10.1186/1477-5956-12-11 (PMC3936806; doi:10.1186/1477-5956-12-11)
Supplement: Additional file 2: Table S2 — Proteomic analysis of PM fraction isolated from brain cortex of control and morphine-treated rats. [file 1477-5956-12-11-S2.doc]

**Table 2**

*Proteomic analysis of PM fraction isolated from brain cortex of control and morphine-treated rats.*

| **Spot** | **Accession** | **Protein name** | **Mascot** | **Matched** | **Peptides** | **SC^a^** | **MW^b^** | **pI^c^** | **Change** |
| --- | --- | --- | --- | --- | --- | --- | --- | --- | --- |
|  | **number** |  | **score** | **peptides** |  | **[%]** | **(kDa)** |  | **(fold)** |
| 1 | BASP1_RAT | **Brain acid soluble** | 284.0 | 6 | K.ETPAASEAPSSAAK.A | 47.3 | 21.8 | 4.3 | ↓ 2.1 |
|  |  | **protein 1** |  |  | K.AEGAGTEEEGTQK.E |  |  |  |  |
|  |  |  |  |  | K.KTEAPAAGPEAK.S |  |  |  |  |
|  |  |  |  |  | K.APAPAAPAAEPQAEAPVASSEQSVAVKE.- |  |  |  |  |
|  |  |  |  |  | K.ESEPQAAADATEVK.E |  |  |  |  |
|  |  |  |  |  | K.AGEASAESTGAADGAPQEEGEAK.K |  |  |  |  |
|  |  |  |  |  |  |  |  |  |  |
| 2 | GBB1_RAT | **Guanine nucleotide-** | 676.3 | 13 | R.LFVSGACDASAK.L | 51.5 | 37.4 | 5.6 | ↓ 2.0 |
|  |  | **binding protein** |  |  | R.LLVSASQDGK.L |  |  |  |  |
|  |  | **subunit beta-1** |  |  | K.LIIWDSYTTNK.V |  |  |  |  |
|  |  |  |  |  | K.IYAMHWGTDSR.L |  |  |  |  |
|  |  |  |  |  | R.AGVLAGHDNR.V |  |  |  |  |
|  |  |  |  |  | K.ACADATLSQITNNIDPVGR.I |  |  |  |  |
|  |  |  |  |  | R.VSCLGVTDDGMAVATGSWDSFLK.I + Oxidation (M) |  |  |  |  |
|  |  |  |  |  | K.VHAIPLR.S |  |  |  |  |
|  |  |  |  |  | K.IYAMHWGTDSR.L + Oxidation (M) |  |  |  |  |
|  |  |  |  |  | R.KACADATLSQITNNIDPVGR.I |  |  |  |  |
|  |  |  |  |  | K.LWDVR.E |  |  |  |  |
|  |  |  |  |  | R.QTFTGHESDINAICFFPNGNAFATGSDDATCR.L |  |  |  |  |
|  |  |  |  |  | R.LLLAGYDDFNCNVWDALK.A |  |  |  |  |
|  |  |  |  |  |  |  |  |  |  |
| 3 | KCRB_RAT | **Creatine kinase B-type** | 945.4 | 17 | R.LGFSEVELVQMVVDGVK.L | 55.6 | 42.7 | 5.3 | ↓2.6 |
|  |  | **(EC 2.7.3.2)** |  |  | R.GTGGVDTAAVGGVFDVSNADR.L |  |  |  |  |
|  |  |  |  |  | R.FCTGLTQIETLFK.S |  |  |  |  |
|  |  |  |  |  | K.LAVEALSSLDGDLSGR.Y |  |  |  |  |
|  |  |  |  |  | R.LEQGQPIDDLMPAQK.- + Oxidation (M) |  |  |  |  |
|  |  |  |  |  | K.DLFDPIIEDR.H |  |  |  |  |
|  |  |  |  |  | K.FSEVLKR.L |  |  |  |  |
|  |  |  |  |  | R.HGGYQPSDEHKTDLNPDNLQGGDDLDPNYVLSSR.V |  |  |  |  |
|  |  |  |  |  | K.VLTPELYAELR.A |  |  |  |  |
|  |  |  |  |  | R.LGFSEVELVQMVVDGVK.L + Oxidation (M) |  |  |  |  |
|  |  |  |  |  | K.LLIEMEQR.L Oxidation (M) |  |  |  |  |
|  |  |  |  |  | K.LRFPAEDEFPDLSSHNNHMAK.V + Oxidation (M) |  |  |  |  |
|  |  |  |  |  | K.TFLVWINEEDHLR.V |  |  |  |  |
|  |  |  |  |  | R.DWPDAR.G |  |  |  |  |
|  |  |  |  |  | R.GFCLPPHCSR.G |  |  |  |  |
|  |  |  |  |  | K.LLIEMEQR.L |  |  |  |  |
|  |  |  |  |  | R.LEQGQPIDDLMPAQK.- |  |  |  |  |
|  |  |  |  |  |  |  |  |  |  |
| 4 | AINX_RAT | **Alpha-internexin** | 222.5 | 5 | R.RLPASDGLDLSQAAAR.T | 16 | 56.1 | 5.1 | ↑5.2 |
|  |  |  |  |  | K.KVESLLDELAFVR.Q |  |  |  |  |
|  |  |  |  |  | K.FANLNEQAAR.S |  |  |  |  |
|  |  |  |  |  | R.AQLEEASSAR.A |  |  |  |  |
|  |  |  |  |  | K.KEEEEEEEEEEGASK.E |  |  |  |  |
|  |  |  |  |  |  |  |  |  |  |
| 5 | DPYL2_RAT | **Dihydropyrimidinase-** | 702.0 | 14 | R.SITIANQTNCPLYVTK.V | 39.0 | 62.2 | 5.9 | ↑4.9 |
|  |  | **related protein 2** |  |  | R.MVIPGGIDVHTR.F + Oxidation (M) |  |  |  |  |
|  |  |  |  |  | R.GLYDGPVCEVSVTPK.T |  |  |  |  |
|  |  |  |  |  | R.FQMPDQGMTSADDFFQGTK.A + Oxidation (M) |  |  |  |  |
|  |  |  |  |  | K.IVLEDGTLHVTEGSGR.Y |  |  |  |  |
|  |  |  |  |  | R.GSPLVVISQGK.I |  |  |  |  |
|  |  |  |  |  | K.QIGENLIVPGGVK.T |  |  |  |  |
|  |  |  |  |  | R.IVAPPGGR.A |  |  |  |  |
|  |  |  |  |  | R.ISVGSDADLVIWDPDSVK.T |  |  |  |  |
|  |  |  |  |  | K.VFNLYPR.K |  |  |  |  |
|  |  |  |  |  | R.KPFPDFVYK.R |  |  |  |  |
|  |  |  |  |  | K.QQAPPVR.N |  |  |  |  |
|  |  |  |  |  | R.LAELR.G |  |  |  |  |
|  |  |  |  |  | R.MSVIWDK.A |  |  |  |  |
|  |  |  |  |  |  |  |  |  |  |
| 6 | SIRT2_RAT | **NAD-dependent** | 136.4 | 3 | R.LLDELTLEGVTR.Y | 17.4 | 39.3 | 6.8 | ↑2.5 |
|  |  | **deacetylase sirtuin-2** |  |  | K.APLATPR.L |  |  |  |  |
|  |  |  |  |  | R.EHANIDAQSGSQASNPSATVSPR.K |  |  |  |  |
|  |  |  |  |  |  |  |  |  |  |
| 7 | SYUA_RAT | **Alpha-synuclein** | 445.1 | 6 | K.TKEQVTNVGGAVVTGVTAVAQK.T | 44.3 | 14.5 | 4.6 | ↑2.0 |
|  |  |  |  |  | K.TVEGAGNIAAATGFVK.K |  |  |  |  |
|  |  |  |  |  | K.EQVTNVGGAVVTGVTAVAQK.T |  |  |  |  |
|  |  |  |  |  | K.EGVVHGVTTVAEK.T |  |  |  |  |
|  |  |  |  |  | K.AKEGVVAAAEK.T |  |  |  |  |
|  |  |  |  |  | K.EGVVAAAEK.T |  |  |  |  |
|  |  |  |  |  |  |  |  |  |  |
| 8 | PRDX2_RAT | **Peroxiredoxin-2** | 239.3 | 6 | R.QITVNDLPVGR.S | 27.3 | 21.8 | 5.2 | ↑2.2 |
|  |  |  |  |  | R.SVDEALR.L |  |  |  |  |
|  |  |  |  |  | K.EGGLGPLNIPLLADVTK.S |  |  |  |  |
|  |  |  |  |  | K.NDEGIAYR.G |  |  |  |  |
|  |  |  |  |  | K.SLSQNYGVLK.N |  |  |  |  |
|  |  |  |  |  | R.KEGGLGPLNIPLLADVTK.S |  |  |  |  |
|  |  |  |  |  |  |  |  |  |  |
| 9 | TERA_RAT | **Transitional endoplasmic** | 457.3 | 10 | R.WALSQSNPSALR.E | 16.6 | 89.3 | 5.0 | ↑2.1 |
|  |  | **reticulum ATPase** |  |  | K.NAPAIIFIDELDAIAPK.R |  |  |  |  |
|  |  |  |  |  | K.LAGESESNLR.K |  |  |  |  |
|  |  |  |  |  | R.ELQELVQYPVEHPDK.F |  |  |  |  |
|  |  |  |  |  | R.ESIESEIR.R |  |  |  |  |
|  |  |  |  |  | R.GILLYGPPGTGK.T |  |  |  |  |
|  |  |  |  |  | R.QAAPCVLFFDELDSIAK.A |  |  |  |  |
|  |  |  |  |  | R.LGDVISIQPCPDVK.Y |  |  |  |  |
|  |  |  |  |  | K.EMVELPLR.H + Oxidation (M) |  |  |  |  |
|  |  |  |  |  | R.GGNIGDGGGAADR.V |  |  |  |  |
|  |  |  |  |  |  |  |  |  |  |
| 10 | DHE3_RAT | **Glutamate** | 342.2 | 7 | R.YSTDVSVDEVK.A | 18.8 | 61.4 | 8.8 | ↑2.7 |
|  |  | **dehydrogenase 1,** |  |  | K.YNLGLDLR.T |  |  |  |  |
|  |  | **mitochondrial** |  |  | K.CAVVDVPFGGAK.A |  |  |  |  |
|  |  |  |  |  | R.DDGSWEVIEGYR.A |  |  |  |  |
|  |  |  |  |  | K.HGGTIPVVPTAEFQDR.I |  |  |  |  |
|  |  |  |  |  | R.GASIVEDK.L |  |  |  |  |
|  |  |  |  |  | K.MVEGFFDR.G |  |  |  |  |
|  |  |  |  |  |  |  |  |  |  |
| 11 | SCOT1_RAT | **Succinyl-CoA:3-ketoacid-** | 102.6 | 2 | K.DGSVAIASKPR.E | 7.5 | 56.2 | 9.5 | ↑2.2 |
|  |  | **coenzyme A transferase 1,** |  |  | K.FYTDPVEAVK.D |  |  |  |  |
|  |  | **mitochondrial** |  |  |  |  |  |  |  |
|  |  |  |  |  |  |  |  |  |  |
| 12 | AATM_RAT | **Aspartate** | 705.3 | 13 | R.IAATILTSPDLR.K | 31.4 | 47.3 | 9.8 | ↓ 2.2 |
|  |  | **aminotransferase,** |  |  | R.VGASFLQR.F |  |  |  |  |
|  |  | **mitochondrial** |  |  | R.DAGMQLQGYR.Y + Oxidation (M) |  |  |  |  |
|  |  |  |  |  | K.EYLPIGGLADFCK.A |  |  |  |  |
|  |  |  |  |  | R.FVTVQTISGTGALR.V |  |  |  |  |
|  |  |  |  |  | R.DAGMQLQGYR.Y |  |  |  |  |
|  |  |  |  |  | K.NLDKEYLPIGGLADFCK.A |  |  |  |  |
|  |  |  |  |  | K.MNLGVGAYR.D + Oxidation (M) |  |  |  |  |
|  |  |  |  |  | R.VGAFTVVCK.D |  |  |  |  |
|  |  |  |  |  | K.NMGLYGER.V + Oxidation (M) |  |  |  |  |
|  |  |  |  |  | R.ISVAGVTSGNVGYLAHAIHQVTK.- |  |  |  |  |
|  |  |  |  |  | K.TCGFDFSGALEDISK.I |  |  |  |  |
|  |  |  |  |  | K.MNLGVGAYR.D |  |  |  |  |
|  |  |  |  |  |  |  |  |  |  |
| 13 | UCHL1_RAT | **Ubiquitin carboxyl-** | 219.9 | 5 | K.QFLSETEKLSPEDR.A | 36.8 | 24.8 | 5.0 | ↓ 2.0 |
|  |  | **terminal hydrolase** |  |  | K.LGVAGQWR.F |  |  |  |  |
|  |  | **isozyme L1** |  |  | K.NEAIQAAHDSVAQEGQCR.V |  |  |  |  |
|  |  |  |  |  | R.MPFPVNHGASSEDSLLQDAAK.V + Oxidation (M) |  |  |  |  |
|  |  |  |  |  | R.FSAVALCK.A |  |  |  |  |
|  |  |  |  |  |  |  |  |  |  |
| 14 | PHB_RAT | **Prohibitin** | 476.4 | 11 | R.IYTSIGEDYDER.V | 47.1 | 29.8 | 5.5 | ↑2.2 |
|  |  |  |  |  | R.FDAGELITQR.E |  |  |  |  |
|  |  |  |  |  | R.NVPVITGSK.D |  |  |  |  |
|  |  |  |  |  | K.AAIISAEGDSK.A |  |  |  |  |
|  |  |  |  |  | R.ILFRPVASQLPR.I |  |  |  |  |
|  |  |  |  |  | K.EFTEAVEAK.Q |  |  |  |  |
|  |  |  |  |  | R.VLPSITTEILK.S |  |  |  |  |
|  |  |  |  |  | R.AVIFDR.F |  |  |  |  |
|  |  |  |  |  | R.QVSDDLTER.A |  |  |  |  |
|  |  |  |  |  | K.AAELIANSLATAGDGLIELR.K |  |  |  |  |
|  |  |  |  |  | K.QVAQQEAER.A |  |  |  |  |
|  |  |  |  |  |  |  |  |  |  |
| 15 | COR1A_RAT | **Coronin-1A** | 143.4 | 4 | R.DGALICTSCR.D | 12.1 | 51.0 | 6.0 | ↓ 5.4 |
|  |  |  |  |  | K.ADQCYEDVR.V |  |  |  |  |
|  |  |  |  |  | R.DAGPLLISLK.D |  |  |  |  |
|  |  |  |  |  | R.LDRLEETVQAK.- |  |  |  |  |
|  |  |  |  |  |  |  |  |  |  |
| 16 | SEP11_RAT | **Septin-11** | 112.5 | 2 | K.FESDPATHNEPGVR.L | 10.0 | 49.7 | 6.2 | ↑2.2 |
|  |  |  |  |  | K.AAAQLLQSQAQQSGAQQTK.K |  |  |  |  |
|  |  |  |  |  |  |  |  |  |  |
| 17 | MBP_RAT | **Myelin basic protein S** | 312.6 | 8 | K.YLATASTMDHAR.H + Oxidation (M) | 33.3 | 21.5 | 11.8 | ↓2.5 |
|  |  |  |  |  | R.DTGILDSIGR.F |  |  |  |  |
|  |  |  |  |  | K.YLATASTMDHAR.H |  |  |  |  |
|  |  |  |  |  | K.NIVTPR.T |  |  |  |  |
|  |  |  |  |  | K.GAYDAQGTLSK.I |  |  |  |  |
|  |  |  |  |  | R.HRDTGILDSIGR.F |  |  |  |  |
|  |  |  |  |  | R.SPLPSHAR.S |  |  |  |  |
|  |  |  |  |  | R.FFSGDR.G |  |  |  |  |
|  |  |  |  |  |  |  |  |  |  |
| 18 | RL12_RAT | **60S ribosomal** | 115.6 | 3 | K.EILGTAQSVGCNVDGR.H | 18.8 | 17.8 | 10.3 | ↑ 2.7 |
|  |  | **protein L12** |  |  | K.LTIQNR.Q |  |  |  |  |
|  |  |  |  |  | K.IGPLGLSPK.K |  |  |  |  |

*^a^* Sequence coverage.

*^b^* Theoretical molecular weight.

*^c^* Theoretical isoelectric point.
